# Integrated Breeding Approaches for Ascochyta Blight Resistance in Chickpea

**DOI:** 10.3390/ijms27157006

**Published:** 2026-08-04

**Authors:** Kadir Akan, Duygu Sari, Hatice Sari, Tuba Eker, Pelin Toker, Aya Yeshengaliyeva, Alibek Zatybekov, Yerlan Turuspekov, Bunyamin Tar’an, Cengiz Toker

**Affiliations:** 1 Department of Plant Protection, Faculty of Agriculture, Kırşehir Ahi Evran University, Kırşehir 40200, Türkiye; kadir.akan@ahievran.edu.tr; 2 Department of Field Crops, Faculty of Agriculture, Akdeniz University, Antalya 07070, Türkiye; ekertuba07@gmail.com (T.E.); toker@akdeniz.edu.tr (C.T.); 3 Department of Crop and Soil Sciences, Washington State University, Pullman, WA 99164, USA; hatice.sari@wsu.edu; 4 Department of Agricultural Biotechnology, Faculty of Agriculture, Akdeniz University, Antalya 07070, Türkiye; tptoker@gmail.com; 5 Department of Agronomy, Breeding and Biotechnology, Faculty of Agrobiology, Kazakh National Agrarian Research University, Almaty 050010, Kazakhstan; ayya.yeshengaliyeva@mail.ru; 6 Laboratory of Molecular Genetics, Institute of Plant Biology and Biotechnology, Almaty 050040, Kazakhstan; alibek.zatybekov@gmail.com (A.Z.); yerlant@yahoo.com (Y.T.); 7 Crop Development Centre and Department of Plant Sciences, University of Saskatchewan, Saskatoon, SK S7N 5E7, Canada

**Keywords:** *Ascochyta rabiei*, resistance, genes, genomic selection, marker-assisted selection

## Abstract

Ascochyta blight (AB), caused by the necrotrophic fungus [*Ascochyta rabiei* (Pass.) Labr.], is one of the most destructive diseases of chickpea (*Cicer arietinum* L.), causing yield losses of up to 100% under favorable conditions. The pathogen possesses a heterothallic mating system with two mating-type idiomorphs, MAT1-1 and MAT1-2, which contribute to high genetic diversity and the frequent breakdown of host resistance. This review summarizes current knowledge of AB biology, epidemiology, and management, highlighting recent advances in molecular diagnostics, host–pathogen interactions, and population dynamics. Conventional and modern detection methods, including PCR-based assays, field-deployable diagnostic tools, and high-throughput phenotyping approaches, are discussed. Resistance to AB is genetically complex and predominantly polygenic, involving multiple quantitative trait loci (QTLs), although major resistance genes have also been reported. Genomic tools such as QTL mapping, genome-wide association studies (GWAS), and genomic selection have accelerated the identification of resistance loci and improved the efficiency of chickpea breeding. The potential of wild *Cicer* species as sources of novel resistance alleles is also emphasized. Integrating genetic resistance with effective disease monitoring and management strategies remains essential for sustainable AB control and the development of durable resistant cultivars.

## 1. Introduction

Chickpea (*Cicer arietinum* L.) is an important legume crop within the subfamily Faboideae Rudd (syn Papilionoideae DC.) of the family Fabaceae Lindl., valuable for human nutrition and sustainable agricultural systems [1,2,3]. The high protein content of its seeds (commonly reported at approximately 18–31%), together with a rich mineral and vitamin profile, adds value both as a feed ingredient in livestock production chains and, as a green manure, within agricultural ecosystem services [4,5,6]. Through symbiosis with *Bradyrhizobium* spp., chickpea fixes atmospheric nitrogen and thereby improves soil fertility; moreover, its relative tolerance to drought and mild low temperatures, along with its ability to grow on marginal soils, contributes to diversifying cereal-dominated rotations and reducing fallow areas [7]. It is mostly a self-pollinating species with a diploid genome (2n = 2x = 16) and, based on morphological and agronomic characteristics, is classified into two major groups—desi and kabuli [4,8]. Desi chickpeas, typically bearing small, dark seeds, predominate in South Asia and Africa, whereas kabuli chickpeas, characterized by large, light-colored seeds, are prevalent across the Mediterranean–West Asian belt. Desi chickpeas have pink or blue flowers with anthocyanin, while kabuli chickpeas have white flowers without anthocyanin [8,9]. Owing to its high protein content and adequate micronutrient content, chickpea contributes to global food and nutrition security; recent nutrition and health research continues to underscore its role in human diets [6]. It is cultivated in more than 55 countries, with South Asia, the Turan region, Australia, North America, and the Mediterranean region accounting for the major share of production [10]. In 2024, global dry chickpea production was 16.9 million tons, harvested from 14.1 million hectares, with an average yield of 1195 kg/ha. India leads global production by a wide margin (11 million tons), followed by Australia (2.3 million tons) and Turkiye (0.58 million tons). Time-series data show that global production rose from 7.7 million tons in 1961 to a peak of 17.8 million tons in 2022, indicating a more than twofold increase [10]. Although the average yield improved from 649 kg per hectare in 1961 to 1237 kg per hectare in 2022 [10], it remained far from its potential yield due to biotic and abiotic stresses [11]. The most impactful common biotic stress is ascochyta blight (AB) of chickpeas caused by *Ascochyta rabiei* (Pass.) Labr. [12].

Chickpea, a key cool-season legume, is severely affected by AB, which under cool, humid conditions produces necrotic lesions on all aerial tissues and can cause total crop failure [13]; moderate to severe epidemics are favored by 10–25 °C and leaf wetness exceeding 12 h [14]. The causal agent is *A. rabiei* (syn. *Phoma rabiei* (Pass.) Khune (1979), whose teleomorph is *Didymella rabiei* (Kovatsch.) Arx. (1962) syn. *Mycosphaerella rabiei* Kovatsch. (1936), a haploid, heterothallic ascomycete with two alternate mating types at a single locus [15,16]. Historically, when bicellular spores were not observed on the host, the pathogen was described as *Phyllosticta rabiei* (Pass.) Trotter (1918) [17]; detection of 2–4% uniseptate spores on artificially inoculated plants prompted adoption of the name *A. rabiei*, now the predominant usage despite taxonomic debate [17]. Although long recognized, AB was first formally reported in 1911 from the North-West Frontier Province of India, now in Pakistan [18,19]. As a necrotroph, *A. rabiei* kills host tissue and proliferates on dead material, and the disease now occurs in more than 40 chickpea-growing countries, imposing substantial losses on global pulse markets [9,20]. Yield losses owing to AB in chickpea reach up to 100% under favorable conditions when a susceptible cultivar is sown [21]. Although AB can be controlled with agricultural chemicals, environmental concerns and economic constraints largely restrict their use in many farmers’ fields [22]. The most environmentally friendly, economical, and practical control of AB can be achieved through an integrated management system that includes host-plant resistance and improved agricultural practices. The achievements of these depend on recent deep knowledge of AB. For successful control of the AB, a chronological review of environmentally sensitive, sustainable, and integrated approaches is required. Simultaneously, QTLs and genes that can be reliably used in genome-based marker-assisted selection studies should be reviewed and updated. In this review, we therefore provide an updated and integrated overview of (i) taxonomy of AB, (ii) races, pathotypes and mating types of AB, (iii) diagnosis and identification of AB, (iv) host plants and resistant resources for AB, (v) management of AB, (vi) screening techniques of AB, (vii) genetics and inheritance for resistance to AB, (viii) QTLs and genes for resistance to AB, and (ix) breeding for resistance to AB. The review also combines recent advances in traditional breeding, standardized phenotyping, wild *Cicer* genetic resources, and modern genomic breeding approaches for improving durable resistance to AB in chickpea.

## 2. Taxonomy of *Ascochyta rabiei*

**Figure 1:**
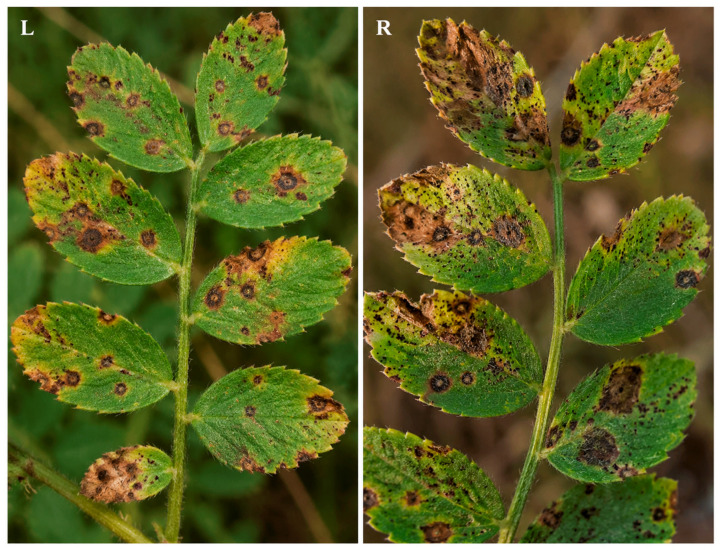
Symptoms of ascochyta blight on leaflets of wild chickpea species ((**Left**): *C. isauricum* P.H. Davis showing typical necrotic lesions with scattered pycnidia, and (**Right**): *C. montbretii* Jaub. & Spach exhibiting more extensive necrotic lesions with abundant pycnidia).

The AB of chickpea is currently accepted as *Ascochyta rabiei* (Pass.). Labr. teleomorph *Didymella rabiei* (Kovatsch.) Arx. (Figure 1), although the early taxonomy of AB was fragmented. Based on hyaline, unicellular pycnidiospores, Passerini (1867) proposed the name *Zythia rabiei*; Comes (1891) allied it with *A. pisi*; and Prillieux and Delacroix (1893) designated it as *Phyllosticta cicerina*. Trotter (1918) showed that the pathogen was not *A. pisi* and more closely resembled *Phyllosticta*, adopting the combination *P. rabiei* (Pass.) Trotter. Subsequently, Labrousse (1931) observed a small proportion of uniseptate spores and consolidated the usage of *A. rabiei* [23]. Several different names were used over time because classifications relied on spore morphology until the current name became established [23]. The sexual and asexual stages were identified. The asexual stage is characterized by erumpent, globose, dark brown to black pycnidia embedded on stems, leaves, pods, and seeds in the host plants, and by hyaline pycnidiospores that are straight to slightly curved, mostly aseptate, and occasionally one-septate [23]. The sexual stage was first detected in 1936 in Bulgaria on overwintered plant debris and named *Didymella rabiei* (Kovatsch.) Arx. (1962), syn. *Mycosphaerella rabiei* Kovatsch. (1936); it was later confirmed on overwintered residues in multiple chickpea-growing regions worldwide [24,25,26]. In this stage, pods bear dark brown to black, beaked, ostiolate pseudothecia; asci are cylindrical to clavate, each containing eight ovoid, two-celled ascospores [9,12]. According to the European and Mediterranean Plant Protection Organization global database [27], the recent taxonomy of AB is given below:

Kingdom: Fungi

Phylum: Ascomycota

Subphylum: Pezizomycotina

Class: Dothideomycetes

Subclass: Pleosporomycetidae

Order: Pleosporales

Family: Didymellaceae

Genus: *Ascochyta*

Species: *A. rabiei* (Pass.) Labr.

## 3. Races, Pathotypes and Mating Type Dynamics of *Ascochyta rabiei*

With respect to pathogenicity, early work on Indian isolates distinguished two principal races (1 and 2) and one biotype; subsequent studies across different differential panels described up to six “pathogenic groups/races” [28,29]. Six races were identified using ICARDA chickpea materials [12,30]. Later studies reported 13 virulent isolates [31]. The sexual reproductive ability of AB to generate new recombinants and/or the continuous emergence of new races through mutations has led researchers to propose alternative classification systems. One of the first DNA-based studies on grouping isolates was conducted by Weising et al. [32]. Subsequent studies have focused on three pathotypes, including pathotype-I, pathotype-II, and pathotype-III, based on reactions to different sets of chickpea differential genotypes [33]. Among these, pathotype III comprised the most virulent isolates [34]. However, direct comparison between race- and pathotype-based classification systems is difficult because different studies have used different differential host sets [18]. Kaiser and Küsmenoğlu(1997) [25] indicated that the teleomorph may contribute to both the long-distance dissemination of the pathogen and the increase in genetic diversity within pathogen populations. Studies using sets of chickpea genotypes based on disease susceptibility, as in the case of races, have identified a fourth pathotype, pathotype-IV [35]. Firouzmand et al. [36] subsequently evaluated resistance against pathotype VI. This continued increase in virulence is often driven by high gene flow and sexual recombination in regions where both MAT1-1 and MAT1-2 mating types coexist in equal frequencies [37]. Such evolutionary pressure necessitates continuous updates to differential sets and global monitoring of isolate aggressiveness to prevent the sudden breakdown of field resistance. The continued identification of additional pathotypes indicates the evolving nature of *A. rabiei*, and pathotype classification remains under discussion. Consequently, race/pathotype definitions have operational importance for pyramiding resistance genes, stabilizing field resistance, and tailoring fungicide strategies to regional populations.

As in other ascomycetes, the sexual cycle is governed by the MAT locus: MAT1-1 encodes an alpha-domain protein, whereas MAT1-2 encodes an HMG-domain DNA-binding protein [38,39]. Mating type is determined by reciprocal pairing with standard tester isolates; distributions of MAT1-1 and MAT1-2 among single-spore isolates from 21 countries and the use of ATCC 76501/76502 as testers have been reported [25]. The nomenclatural history and biphasic life cycle of *A. rabiei* underpin substantial genetic and phenotypic diversity within and among populations. Where the sexual stage is active in agroecosystems, the risk of novel pathotype formation and epidemics increases—making seed health, residue management, and the deployment of resistant cultivars primary priorities. The reproductive biology of *Ascochyta rabiei* is governed by a heterothallic mating system defined by two distinct idiomorphs, MAT1-1 and MAT1-2, which encode distinct regulatory proteins [25,40]. MAT1-1 produces a DNA-binding protein with an α-domain, while MAT1-2 encodes a protein containing a high mobility group (HMG) domain [39,41]. The coexistence of MAT1-1 and MAT1-2 within a population is essential for the completion of the sexual cycle, which, in turn, has significant consequences for the pathogen’s genetic diversity and epidemiology [25,42].

In ascomycete fungi, the MAT locus is the central regulator of sexual reproduction, and species may exhibit either heterothallic (self-incompatible) or homothallic (self-compatible) reproductive strategies. In heterothallic species like *A. rabiei*, MAT1-1 and MAT1-2 are present in separate nuclei, whereas homothallic species possess idiomorphs within a single nucleus [43]. The presence of both mating types in a population enables sexual recombination, which can generate novel aggressive races with altered pathogenicity and virulence, potentially resulting in the emergence of more aggressive strains [42,44].

Advances in molecular tools, such as MAT-specific multiplex PCR assays, have enabled rapid and reliable differentiation between MAT1-1 and MAT1-2 isolates [45]. Certain conserved regions within the MAT loci are also valuable for phylogenetic studies [46].

### 3.1. Chronological Overview of Mating Type Distribution Studies

The distribution of mating types has been evaluated in several chickpea-growing regions over the past three decades. In Turkey, both MAT1-1 and MAT1-2 were detected, suggesting the potential for sexual reproduction [25]. Similar distributions were subsequently reported in Canada [26], Pakistan and the United States [47], Syria [48], and Morocco [37], where the coexistence of both mating types indicates an increased potential for genetic recombination and pathogen diversification.

### 3.2. Synthesis and Implications

The widespread occurrence of both mating types highlights the evolutionary potential of *A. rabiei*. Sexual recombination can lead to more virulent strains, posing ongoing challenges for chickpea disease management. Therefore, continuous monitoring and molecular characterization of mating type distributions are crucial for understanding the reproductive biology and epidemiological dynamics of this important pathogen.

## 4. Diagnosis and Identification of *Ascochyta rabiei*

### 4.1. Advances and Challenges in the Diagnosis of AB in Chickpea

Effective management of AB in chickpeas depends on accurate and timely diagnosis. Traditionally, field diagnosis is based on observing disease symptoms, which typically become apparent only at moderate to advanced stages of infection. This symptom-based approach, while useful for large-scale monitoring, is limited by its inability to detect early or latent infections and often requires significant expertise [49,50]. Laboratory confirmation is based on culture and microscopic examination. However, these methods are time-consuming and often require specialized skills. Molecular diagnostic techniques have been developed to overcome these challenges, offering greater sensitivity and specificity. PCR-based assays targeting unique fungal DNA sequences, such as the internal transcribed spacer (ITS) regions, are particularly valuable for detecting *A. rabiei* in asymptomatic or low-biomass samples. However, ITS alone may not clearly distinguish closely related *Ascochyta* species. Therefore, additional molecular markers may improve identification accuracy. Further refinement in molecular diagnostics includes sequence-characterized amplified region (SCAR) markers and β-tubulin gene assays. Despite the reliability of conventional PCR in laboratory settings, its application in field diagnostics is limited, especially in regions lacking advanced infrastructure; therefore, isothermal amplification methods such as loop-mediated isothermal amplification (LAMP), which can be performed with minimal equipment and expertise, have been introduced as rapid, on-site diagnosis [51].

Emerging technologies continue to push the boundaries of plant pathogen detection. Recombinase polymerase amplification (RPA) has strong potential for future use due to its rapid, sensitive, and field-adaptable DNA amplification [18,52].

Integrating nanotechnology with molecular diagnostics is expected to further enhance the portability, sensitivity, and flexibility of detection systems, thereby supporting more effective future disease management strategies [18].

Furthermore, recent advances in digital agriculture, including machine-learning-based image analysis and drone-assisted multispectral imaging, offer new opportunities for early, large-scale detection of AB. These approaches can complement molecular diagnostics and enable real-time disease monitoring.

### 4.2. Pathogenic Mechanisms and Survival Strategies of A. rabiei

*A. rabiei*, a necrotrophic fungus, demonstrates a remarkable ability to persist in agricultural environments, primarily due to its capacity to survive in crop residues and seeds for extended periods [53]. The fungus can remain dormant in plant debris for up to four years, which allows it to bridge the gap between growing seasons even without a suitable host. Notably, chickpea seeds serve as a crucial reservoir for *A. rabiei*, as the pathogen can persist on the seed coat, cotyledons, and embryo for over five months, facilitating both its survival and dissemination across fields and seasons [53].

The infection typically begins when *A. rabiei* conidia land on the surface of chickpea plants, particularly during the flowering and pod development stages [54,55]. Upon contact, the spores germinate and produce germ tubes that develop appressorium-like structures, enabling the fungus to adhere to and penetrate the plant epidermis [55].

Early symptoms of infection are characterized by small, water-soaked lesions on leaves, which later turn brown and may develop into concentric rings or elongated lesions on stems and petioles [55], potentially leading to the collapse and death of the entire plant, although high temperatures may limit disease progression [53]. Seed infection is important because infected seeds can serve as a primary source of inoculum [56].

### 4.3. Molecular Mechanisms of Host–Pathogen Interaction

Beyond physical symptoms, the interaction between *A. rabiei* and chickpea involves complex molecular reprogramming. Recent transcriptomic studies indicate that resistant genotypes initiate a rapid defense response within 24 to 72 h post-infection [18,57]. This includes the upregulation of genes involved in cell wall remodeling (e.g., lignin and suberin biosynthesis) and the activation of phytohormone signaling pathways, specifically jasmonic acid (JA) and ethylene (ET) [58]. Identifying these ‘hub genes’, such as those found near the AB4.1 QTL, provides a molecular blueprint for developing cultivars with more durable, multi-layered resistance [59].

In conclusion, understanding these mechanisms at the molecular level is essential for devising effective management strategies against AB in chickpea cultivation.

## 5. Host Plants and Resistant Resources for AB

Chickpea is the most common host of *A. rabiei*, making it highly susceptible to AB on chickpea residue under cool (5–10 °C) and moist conditions (typically winter/early spring) [56]. It infects all aboveground parts of the chickpea plant, including leaves, petioles, stems, pods, and seeds. One of the primary objectives of breeding efforts is to enhance the resistance of newly developed commercial cultivars to this pathogen [60].

*A. rabiei* can infect and occasionally colonize other plant species, such as pea, lentil, and alfalfa, but typically with less severe symptoms than on chickpeas. These alternative hosts may contribute to the fungus’ survival under certain conditions, although natural cross-infection is considered rare [61].

Not only cultivated chickpeas but also annual wild chickpeas are considered a valuable genetic resource for resistance to AB. Robertson et al. [62] evaluated 228 accessions of eight wild *Cicer* species for resistance to AB and selected 17 accessions of *C. pinnatifidum* Jaub. & Sp., nine accessions of *C. bijugum* K.H. Rech., four accessions of *C. judaicum* Boiss. and an accession of *C. echinospermum* P.H. Davis. Following the aforementioned screening studies, Benzohra et al. [63] reported that two accessions of *C. echinospermum*, three accessions of *C. reticulatum*, and all accessions of *C. judaicum* were resistant to AB isolates.

A comprehensive collection mission was initiated a decade ago since the limited number of *C. echinospermum* and *C. reticulatum* exist in gene banks [64], and new genetic sources for resistance to AB were reported in some accessions of *C. biugum* and *C. echinospermum* [65], a few accessions of *C. echinospermum* and *C. reticulatum* in Australia and Turkiye [66]. Pande et al. [67] comprehensively reviewed genetic resources for resistance to AB. Not only annual wild *Cicer* species but also perennials possess valuable genetic resources [68]. C. *reticulatum* and *C. echinospermum* are crucial to get rid of the bottleneck in cultivated chickpeas because they are crossable with cultivated chickpeas [69]. Notably, wild species play a crucial role in the search for resistance to pathogens. *A. rabiei* is a host-specific pathogen of *Cicer* species under natural conditions, meaning that other legumes—such as lentils, peas, or beans—are not typical hosts. Consequently, cross-infection under natural conditions is rare or does not occur. This host specificity is important for breeding since resistance genes can be introgressed from wild *Cicer* species [70].

*A. rabiei* resistance is highly genotype-dependent, underscoring the significance of *Cicer* varietal variation in breeding initiatives to enhance disease resistance. The variations in AB resistance among *Cicer* species that have been discovered highlight how innate genetic traits—especially those present in wild species—can modify plant–pathogen interactions.

Compared to other legume–Ascochyta pathosystems (e.g., pea and lentil), chickpea exhibits a narrower genetic base for resistance, making it more vulnerable to pathogen evolution.

## 6. Management of AB

Managing AB remains challenging due to the scarcity of cultivars with robust resistance and the lack of fungicides that provide reliable protection. Moreover, environmental conditions often favor the rapid spread and persistence of the disease, complicating management efforts [71]. Generally, the available strategies integrate agronomic practices, the use of targeted fungicides, host resistance and the employment of biological agents for disease suppression [72].

### 6.1. Redefining Cultural Approaches in AB Suppression

Cultural strategies primarily aim to minimize the presence of infectious agents in the field. Ensuring certified, disease-free seeds is crucial, as it substantially lowers the likelihood of introducing seed-borne pathogens into new crops [73].

Rotating chickpeas with non-host crops, such as cereals, is another effective measure to decrease the residual inoculum in the soil. Adjusting the timing of sowing can help limit plants’ exposure to infectious spores, thereby reducing disease incidence [73]. Additionally, increasing row spacing and selecting cultivars with upright, compact growth can create a less favorable microclimate for the pathogen by lowering humidity and moisture accumulation [74].

Applying balanced fertilizers, particularly supplementing potassium in nitrogen-rich soils, has been shown to bolster chickpea resilience [9]. When these cultural interventions are implemented collectively and systematically, they can significantly reduce disease pressure on chickpea crops [75].

### 6.2. Harnessing Genetic Resistance in Chickpea for Sustainable AB Control

Leveraging the genetic diversity within chickpea populations has emerged as a pivotal strategy for combating AB sustainably and cost-effectively [76]. To broaden the genetic base for resistance, attention has increasingly turned to wild relatives of chickpeas, which often harbor unique genes conferring resilience to biotic stresses [77]. The integration of these genes into breeding programs is facilitated by a range of screening techniques, including field trials, greenhouse assays, and innovative methods such as fogger irrigation and mini-dome tests [9,78].

Advances in genomics are accelerating the identification of morphological, biochemical, and molecular traits for use in marker-assisted selection [79,80,81,82]. However, achieving durable, broad-spectrum resistance remains challenging.

### 6.3. Expanding the Role of Biological Control in AB Suppression

Biological control has gained increasing attention as an environmentally responsible alternative to chemical interventions. A variety of biocontrol agents, such as *Trichoderma, Aureobasidium, Burkholderia*, *Pseudomonas* and *Rhizobium,* have been reported to significantly reduce pathogen growth and disease symptoms (Table 1) [83]. The mechanisms by which these biocontrol agents operate are diverse: they may outcompete pathogens for resources, parasitize or directly attack the pathogen, secrete lytic enzymes or antibiotics, or trigger systemic resistance responses in the host plant [84,85].

Beyond microbial agents, plant-derived substances such as botanical extracts and essential oils are also being investigated for their antifungal properties. For example, extracts and oils from different plants have shown potential for suppressing *A. rabiei* and other plant pathogens [93,94,95].

Despite these advances, the effectiveness of biological control agents can be inconsistent, as their activity is often influenced by environmental factors, and their practical application requires further research and optimization to ensure consistent efficacy under diverse agricultural conditions.

### 6.4. Optimizing Chemical Approaches for AB Control: Efficacy, Resistance, and Future Directions

Chemical interventions remain a vital part of AB management, especially in the absence of highly resistant chickpea cultivars and due to the disease’s ability to cause rapid outbreaks under favorable conditions [9,96,97,98]. Especially, systemic fungicides such as benomyl, thiram, carbendazim, and chlorothalonil have been shown to significantly reduce seed-borne transmission [99]. Thiabendazole-based treatments and their combinations also effectively limit the pathogen’s spread through contaminated seeds [100]. However, the success of these treatments in the field is influenced by environmental factors.

Despite their proven efficacy, fungicides are associated with high costs and potential risks to human health and the environment, including contamination of food and ecosystems [73,101]. This has driven interest in alternative solutions, such as plant-derived extracts and essential oils [92,102]. In addition, repeated use of fungicides may reduce their effectiveness due to the development of fungicide resistance in *A. rabiei* [101]. Therefore, fungicides should be used as part of an integrated disease management strategy.

Advances in disease forecasting models have also improved the precision and timing of fungicide applications. Integrated models that account for weather, pathogen biology, and crop growth stage have enhanced the accuracy of management recommendations [103,104].

### 6.5. Holistic Approaches to Integrated Disease Management of AB in Chickpea

Integrated disease management (IDM) is now recognized as the most effective approach for managing AB [105] and combines cultural, chemical, and genetic strategies to maximize disease suppression and crop productivity.

Climate change is expected to significantly influence the epidemiology of AB by altering temperature and humidity regimes, potentially expanding the geographic range and increasing the frequency of epidemics [104,106]. Therefore, the future of chickpea protection lies in a ‘Genomics-Assisted IDM’ framework [107]. This involves integrating genetically resistant cultivars—developed through gene pyramiding and genomic selection (GS)—with real-time disease forecasting models and the strategic use of biological agents like *Trichoderma* and *Rhizobium* [108]. Such a holistic approach reduces reliance on chemical fungicides, mitigates the risk of pathogen resistance to strobilurins, and ensures the long-term sustainability of chickpea production systems [101].

## 7. Screening Approaches

Effective screening for AB resistance is an important part of chickpea breeding. It depends on reliable, repeatable, and scalable phenotyping methods [9]. Traditional screening methods are generally performed under both field and controlled conditions. The whole-plant screening technique, cut-twig screening technique, and detached-leaf technique were significantly associated with the greenhouse and field screening techniques [9]. Field screening commonly includes planting test genotypes alongside susceptible cultivars (ILC 263 and ICC 4991) as indicator lines. Susceptible checks are usually planted after every 2–4 entries to maintain uniform inoculum distribution throughout the experimental plot [9,21,23]. The screening nursery must include at least an AB-resistant genotype to compare test lines/genotypes. Disease screening of the genotypes was assessed three times at the seedling, flowering, and pod-setting stages [21]. After inoculation, the genotypes were screened using the 1–9 visual rating scale originally developed by Reddy and Singh [109] and later used by Pande et al. [9] and Chen et al. [78]. The scale is defined as follows: 1 = immune, no infection; 2 = highly resistant, 1–5%; 3 = resistant, 6–10%; 4 = moderately resistant, 11–15%; 5 = tolerant, 16–40%; 6 = moderately susceptible, 41–50%; 7 = moderately susceptible to susceptible, 51–75%; 8 = susceptible, 76–100% breakage of branches and pod infection; and 9 = highly susceptible, plants killed.

PCR-based techniques, including RT-PCR, are fast, accurate, and precise methods for pathogen identification and are widely used in epidemiological studies [110].

Recent advances in high-throughput phenotyping have led to more effective disease management. Among these, sensors provide valuable data and information on disease development [111]. Consequently, accurate and reliable phenotyping plays a critical role in genomic studies. Modern approaches such as QTL mapping, GWAS, and marker-assisted selection (MAS) depend on precise phenotypic data. This indicates the importance of effective screening in disease management.

## 8. Genetics of AB Resistance

Understanding the inheritance of resistance is vital for advancing breeding programs and developing durable cultivars. Early genetic studies revealed that resistance to *A. rabiei* can be governed by either dominant or recessive genes, depending on the source of resistance. Singh and Reddy [112] evaluated disease severity in F_2_ populations derived from 12 cross combinations using a 1–9 rating scale, revealing simple Mendelian inheritance. Their results showed that resistance was controlled by either a single recessive gene (*rar* 1) or a dominant gene (*Rar* 2). These early insights laid the foundation for mapping and breeding efforts using resistant lines from both desi and kabuli backgrounds.

Further studies have shown that the inheritance of resistance may be more complex [113]. In particular, the RIL population exhibited a 1:7 [resistant (R): susceptible (S)] segregation ratio, indicative of resistance governed by three complementary recessive genes. The results underscored the quantitative and multigenic nature of resistance, further modulated by genetic background and pathotype specificity.

Subsequent studies confirmed that AB resistance is often polygenic and strongly influenced by environmental and pathotype variation. Bhardwaj et al. [114] conducted a comprehensive inheritance study using multiple generations (P_1_, P_2_, F_1_, F_2_, F_3_, BC_1_, and BC_2_) from diverse crosses involving resistant and susceptible chickpea lines. In susceptible × susceptible crosses such as GL 769 × C 214, F_2_ progeny exhibited a 15 (susceptible):1 (resistant) segregation ratio, indicating the presence of two complementary recessive genes, each parent carrying a weak resistance gene that is only effective when homozygous and combined. In resistant × susceptible crosses, varying inheritance patterns emerged. For instance, GL 769 × GG 1267 and C 214 × GG 1267 showed 13:3 (R:S) ratios, suggesting control by one dominant and one recessive gene, while GL 769 × GL 90168 fit a 3:1 ratio, supporting monogenic dominant inheritance. Interestingly, C 214 × GL 90168 displayed a 49:15 (R:S) segregation ratio, indicating trigenic control with at least one dominant and two recessive genes involved. Similarly, crosses with GL 96010 revealed both monogenic and trigenic inheritance patterns, depending on the susceptible parent used. In contrast, crosses with GL 98010 showed a 1:3 (R:S) segregation ratio, confirming monogenic recessive control of resistance. Resistant × resistant crosses largely exhibited allele-dominant resistance genes, though some combinations (e.g., GL 90168 × GL 98010) segregated 13:3, suggesting non-allelic gene interactions. These results indicate a complex genetic architecture involving major dominant and recessive genes, minor modifiers, and inhibitory alleles that contribute to AB resistance in chickpeas.

Recent studies reinforce the idea that resistance is quantitatively inherited and pathotype-specific, differing in aggressiveness and virulence, emphasizing the need for screening across multiple environments and pathogen races to achieve stable resistance [21,115,116,117]. Environmental factors such as temperature, humidity, and plant developmental stage can alter disease expression, contributing to genotype-by-environment (G × E) interactions [53]. Udupa and Baum [115] conducted a study to unravel the genetic basis of pathotype-specific resistance to *A. rabiei* in chickpea using a RIL population derived from a cross between ILC 3279 (resistant) and ILC 1272 (susceptible). They reported that resistance to pathotype-I is governed by a single recessive gene (*ar1*) mapped to linkage group 2 (LG2). In contrast, resistance to pathotype-II is controlled by two independent recessive genes, *ar2a* and *ar2b*, which show complementary gene action and are located on LG2 and LG4, respectively. Interestingly, *ar2a* was mapped near the *ar1* locus, suggesting a genomic cluster of resistance genes. These findings demonstrate clear genetic differentiation in resistance mechanisms among pathotypes and underscore the importance of precise phenotyping and controlled-environment screening in resistance mapping. These insights into pathotype-specific resistance loci provide a foundational framework for dissecting the genetic control of *A. rabiei* resistance in cultivated chickpea. However, to further broaden the resistance gene pool, researchers have turned to wild *Cicer* species [66,117,118,119], which offer untapped reservoirs of genetic diversity.

Wild relatives such as *C. reticulatum* and *C. echinospermum* harbor unique alleles for disease resistance. Lakmes et al. [120] evaluated wide crosses involving these wild species and found broad segregation for AB resistance, with transgressive segregation indicating novel allele combinations. Their study identified four QTLs linked to early and late resistance responses: a QTL on chromosome 7 in the *C. reticulatum* crosses was associated with late-stage resistance. Three QTLs on chromosomes 2, 3, and 6 in the *C. echinospermum* crosses were linked to early-stage resistance. Most wild alleles were associated with reduced disease severity, while heterozygotes often exhibited intermediate or higher susceptibility, indicating additive and dominance effects [120]. These findings highlight the breeding potential of wild gene pools, which remain underexploited in elite cultivars.

The genetic architecture of AB resistance in chickpea, spanning monogenic to polygenic, additive to epistatic, demands tailored breeding approaches. For simply inherited traits, marker-assisted backcrossing can be effective. For complex resistance, QTL pyramiding, genomic selection (GS), and multi-parent advanced generation intercross (MAGIC) populations provide valuable tools for accumulating minor alleles that contribute to durable resistance. The evidence from wide crosses and diverse germplasm highlights the necessity of exploiting wild relatives to identify novel alleles, avoiding susceptible backgrounds harboring inhibitory loci, incorporating multi-pathotype screening, and validating resistance in multi-environment trials. Molecular approaches, including QTLs, SNPs, and candidate genes associated with AB resistance, are discussed in detail in the following section.

## 9. QTLs and Genes for Resistance to AB

Advances in molecular markers and bioinformatics have enabled more effective study of the complex genetic basis of resistance traits. These tools have helped identify quantitative trait loci (QTLs) that contribute to resistance against AB. Today, AB resistance in chickpea is understood as a quantitative trait controlled by multiple QTLs [81]. Over the past two decades, many QTLs linked to AB resistance have been mapped across different linkage groups using various genetic markers. These markers have made it easier and faster to develop resistant genotypes by combining resistance genes from different sources.

Early research on AB resistance in chickpea focused on genes with strong phenotypic effects. However, Santra et al. [121] were the first to classify AB resistance as a quantitative trait, identifying two QTLs (QTL1 and QTL2) in a recombinant inbred line (RIL) population derived from *C. arietinum* (FLIP84-92C, resistant parent) × *C. reticulatum* (PI 599072, susceptible parent). (Table 2). These QTLs explained 50% and 45% of phenotypic variation. Subsequent work by Tekeoglu et al. [122] added STMS markers (GAA47, TA72, TA2, TS54) to refine the linkage map, demonstrating additive effects between the two QTLs and confirming their location on LG 1 and 6, respectively.

The pathogenicity of different *A. rabiei* pathotypes can affect disease resistance [34]. Pathotype-specific resistance has been investigated in many biparental populations. Udupa and Baum [115] studied a major resistance locus *ar1*, on LG2 for pathotype-I, and two independent recessive loci (*ar2a* on LG2 and *ar2b* on LG4) for pathotype-II. Consistent with these findings, Cho et al. [116] identified two major QTLs on LG2 for pathotype-I and LG4 for pathotype-II using the chickpea linkage map developed by Winter et al. [150]. In addition, the SSR marker TA46 on LG2 showed a strong association with pathotype-II resistance derived from the FLIP84-92C line, explaining 59–69% of the phenotypic variation under controlled conditions [116]. Similarly, Ilyas et al. [151] reported that a single putative resistance gene, *Ar19*, conferred resistance to *both A. rabiei* pathotypes, with strong resistance to pathotype-I and partial resistance to pathotype-II. Collectively, these studies consistently highlight LG2 and LG4 as the principal genomic regions controlling pathotype-specific resistance. Subsequent mapping studies identified additional QTLs associated with AB resistance on LG2, LG3, LG4, LG6, and LG8 using different biparental populations and marker systems [128,135,136,152,153,154,155]. Although resistance-associated loci have been reported across multiple linkage groups, LG2 and LG4 have been the most consistently identified resistance regions across independent studies. In particular, QTLs on LG2 were repeatedly associated with major resistance effects [115,116,125,128], whereas LG4 appears to harbor a cluster of resistance loci, supporting its importance for marker-assisted breeding [123,126,128,153,154]. In addition, markers such as CaETR [134] have been developed from these stable QTL regions and have practical utility in breeding programs to help eliminate susceptible genotypes. Stephens et al. [135] further identified two QTLs on LG4, including one previously reported QTL (ab_QTL1) [115,128,153,154] and one novel QTL (ab_QTL2). Later, Daba et al. [136] identified eight QTLs distributed across all linkage groups except LG5 using SNP markers. In summary, a total of 14 *Ar* resistance loci have been identified across eight chickpea linkage groups using traditional biparental populations, highlighting their potential value for marker-assisted breeding [115,116,123,126,127,128,129,133,135,153,154,155].

Recent advances have identified several candidate genes potentially associated with AB resistance belonging to defense-related families, including WRKY transcription factors, NBS-LRR proteins, and receptor-like kinases [57,156]. Given the necrotrophic nature of *A. rabiei*, resistance is often associated with jasmonic acid and ethylene signaling pathways rather than salicylic acid-mediated responses [18]. Integrating QTL mapping with transcriptomic and proteomic analyses is increasingly necessary to identify causal genes and regulatory networks [79].

While bi-parental mapping has identified over 14 *Ar* resistance loci, GWAS integration has narrowed these regions to specific candidate genes, including a 100 kb interval on LG 4 [137]. Furthermore, GS models now enable prediction of genomic estimated breeding values (GEBVs) for AB resistance across entire germplasm collections [107,145]. This allows for the selection of superior lines based on genomic data alone, significantly reducing the reliance on multi-year, multi-location field phenotyping, which is often confounded by environmental interactions.

## 10. Breeding for Resistance to AB

**Figure 2:**
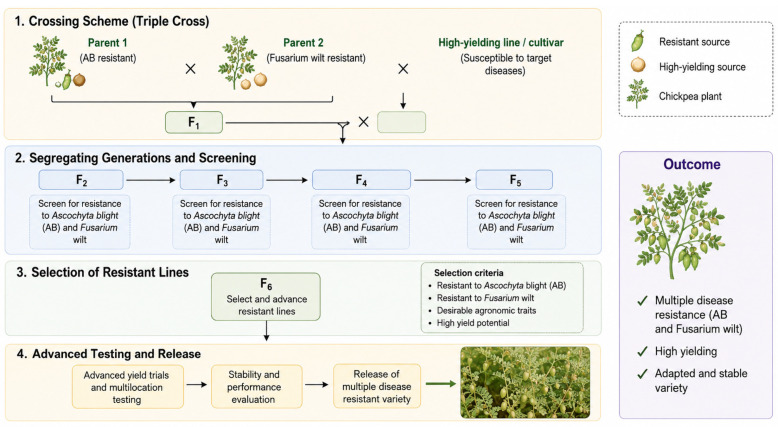
Selection for resistance to AB and fusarium wilt in alternative generations.

The known breeding methods for self-pollinated crops have been effectively used [157]. These include the pedigree, bulk, modified bulk, single-seed descent (SSD), stepwise breeding, recurrent selection in self-pollinated crops, and mutation breeding methods, including polyploidy and induced mutations. Depending on the breeding objective, single, double, and triple crosses have also been used [157,158,159,160]. Interspecific crosses in chickpea have been advised for yield and quality traits, while intraspecific crosses in *Cicer* species have been suggested for resistance to a/biotic stresses [69]. Porta-Puglia et al. [158] showed stepwise breeding for multiple resistances, including AB and fusarium wilt, in chickpea using triple crosses. F_1_ plants from two resistant parents are crossed with a high-yielding line or cultivar. The progeny were screened for resistance to two biotic stresses from F_2_ to F_5_, and the selected resistant lines were maintained in F_6_ (Figure 2).

To combat the rapid evolution of *A. rabiei*, breeding programs are transitioning toward ‘Gene Pyramiding.’ By stacking multiple minor quantitative trait loci (QTLs) from both cultivated and wild *Cicer* gene pools, breeders can achieve more durable resistance. For example, combining the major-effect QTL from LG 4 (AB4.1) with late-responding QTLs on chromosome 7 of *C. reticulatum* has the potential to confer a broad-spectrum defense [139,147]. This approach is increasingly streamlined by marker-assisted backcrossing (MABC), which uses specific SNP markers, such as CaETR, to eliminate susceptible plants in early generations [14,161].

**Figure 3:**
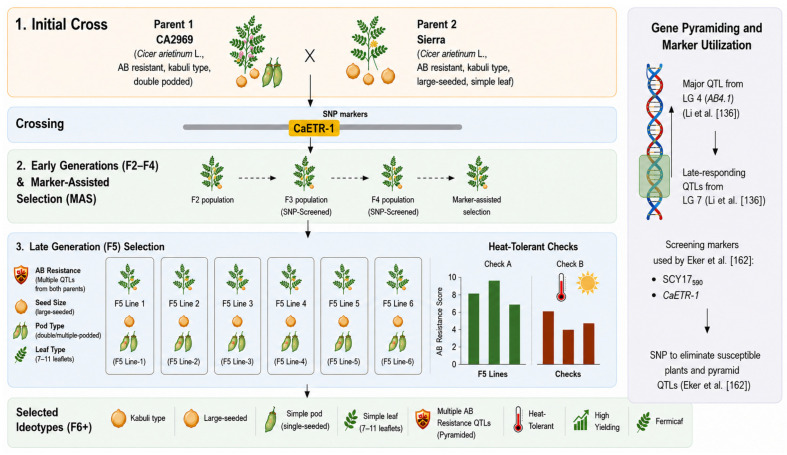
Selection for resistance to AB and heat tolerance in kabuli chickpea.

Eker et al. [162] explained how kabuli chickpea ideotypes were selected. Two genetically different chickpea cultivars with different QTLs for resistance to AB were crossed, and six lines in F_5_ were selected for high yield, having two QTLs, double-podded or multiple-podded, with fern leaves, and larger seeds (heavier than 50 g per 100 seeds). These lines not only had attractive agronomic and morphological traits but were also identified as heat-tolerant based on comparisons with heat-tolerant checks (Figure 3).

Emerging approaches such as GS enable the prediction of breeding values using genome-wide markers, allowing more efficient accumulation of minor-effect resistance loci [107]. Integration of advanced genomic approaches with high-throughput phenotyping and genotyping platforms is expected to accelerate the development of durable AB-resistant cultivars [107,142].

In summary, the combination of traditional breeding, molecular techniques, and genomic technologies plays an important role in breeding for resistance to AB. Future breeding efforts should focus on integrating resistance loci from cultivated and wild *Cicer* species to provide long-term control of AB. Using advanced phenotyping technologies will also be essential for effective disease control and ensuring the sustainability of chickpea production.

## 11. Conclusions

Effective management of AB depends on accurate diagnostics, a better understanding of pathogen biology and population diversity, and reliable disease screening. These advances will support the development of chickpea cultivars with durable resistance by integrating resistance sources from cultivated and wild *Cicer* species with advanced genomic tools. Together, these approaches will help achieve sustainable disease management and chickpea production.

## Figures and Tables

**Table 1:** Examples of Biological Agents Effective Against *A. rabiei.*

Type of Agent	Name	Activity Against *A. rabiei*	Evaluation	Reference
Fungal biocontrol agents	*Chaetomium globosum* Cg2	Inhibited mycelium growth	In vitro	[86]
	*Trichoderma viride* TV-5-2	Suppressed growth and sporulation	In vitro	[86]
	*Acremonium implicatum* (Isolate 1)	Inhibited and lysed mycelium	In vitro	[86]
	*Acremonium implicatum* (Isolate 2)	Inhibited mycelium growth after 7 days	In vitro	[87]
	*Trichoderma harzianum*	Produced chitinase and β-1,3-glucanase	In vitro	[88]
	*T. harzianum* T15	Reduced lesions on plants	Greenhouse	[89]
	*Aureobasidium pullulans*	Inhibited mycelium (>30%)	In vitro	[90]
Bacterial biocontrol agents	*Pseudomonas fluorescens*	Inhibited mycelium and disease development	In vitro + Greenhouse	[53]
	*Pseudomonas putida*	Inhibited spore germination	In vitro	[91]
	*Burkholderia multivorans*	Reduced fungal biomass (70%)	Greenhouse	[92]
	*Mesorhizobium ciceri*	Inhibited mycelium growth	In vitro	[86]
	*Burkholderia ambifaria*	Suppressed growth and sporulation	In vitro	[86]
	*Burkholderia ambifaria*	Inhibited and lysed mycelium	In vitro	[86]
	*Bacillus megaterium*	Inhibited mycelium growth after 7 days	In vitro	[87]
Botanical extract	*Chenopodium album*	Produced chitinase and β-1,3-glucanase	In vitro	[92]

**Table 2:** List of AB-resistant QTLs/Genes identified in chickpea.

Mapping Approach	Population/Germplasm	Marker	QTL/Gene	Chromosome (Ca)/Linkage Group (LG)	PVE (%)	Reference
Bi-parental	FLIP84-92C × *C. reticulatum* Lad. (PI 599072)	RAPD, ISSR	QTL-1 and QTL-2	LG1, LG6	45–50.30	[121]
Bi-parental	Lasseter × *C. echinospermum* (PI 527930), F_2_	STMS	1 QTL	LG4	NR	[123]
Bi-parental	ILC 1272 × ILC 3279	SSR	*ar1*, *ar2a*, *ar2b*	LG4, LG2	NR	[115]
Bi-parental	PI 359075 × FLIP 8492C (F_7_ RIL)	SSR	3 QTL, Ar19 gene	LG4A, LG2, LG6	NR	[116]
Bi-parental	*C. arietinum* (ILC 72) × *C. reticulatum* (Cr5-10)	RAPD, ISSR, STMS, Isozymes	1 QTL	LG2	28	[124]
Bi-parental	ILC 3279 × WR 315 (F_6:7_ RIL)	STMS	QTLAR3	LG2	11.3–22.6	[125]
Bi-parental	‘ICCV 96029′ ‘CDC Frontier’ (186 F_2_)	SSR	3 QTL	LG3, LG4, LG6	12–29	[126]
Bi-parental	CDC Frontier × ICCV 96029, CDC Luna × ICCV 96029, CDC Corinne × ICCV 96029, Amit × ICCV 96029, F_1_ and F_2_	SSR	5 QTL	LG2, LG3, LG4, LG6 and LG8	14–56	[127]
Bi-parental	ICC 4991 × ICCV 04516	SSR	3 QTL	LG3, LG4	7.7–18.6	[128]
Bi-parental	ICC 3996 (*C. arietinum*) × ILWC 184 (*C. reticulatum*)	SSR	3 QTL	LG3, LG4	49	[129]
Bi-parental	ILC 3279 × WR 315, WR 315 × ILC 3279, F_6:7_ RIL	SSR	QTLAR1, CaETR-1 sequence	LG4	33.8	[130]
Backcross	CDC Xena × CDC Frontier, CDC Xena × CDC 425-14	SSR	*Abr QTL3*, *Abr QTL4*	LG4, LG8	NR	[131]
Bi-parental	C 214 × ILC 3279 (F2:3)	SSR	AB-Q-SR-4-1, AB-Q-SR-4-2, AB-Q-APR-6-1, AB-Q-APR-6-2, AB-Q-APR-4-1, AB-Q-APR-5B	LG4, LG5, LG6	1.5–31.9	[132]
Bi-parental	ILC 72 (*C. arietinum*) × Cr5-10 (*C. reticulatum*) (F_6:7_ RIL)	STMS, Genic molecular marker, ETS	42 candidate genes, *Ein3*, *Avr9/Cf9* and *Argonaute 4*	Ca2	44.3	[133]
Bi-parental	Lasseter × ICC 3996, S95362 × Howzat	EST-SSR, SNP	ab_QTL1, ab_QTL2	LG4	14–45	[134]
Bi-parental	ICCV 96029 × CDC Frontier (92 RIL)	SNP	qtlAb-1.1, qtlAb-2.1, qtlAb-3.1, qtlAb-4.1, qtlAb-6.1, qtlAb-7.1, qtlAb-8.1, qtlAb-8.2, qtlAb-8.3	LG1, LG2, LG3, LG4, LG6, LG7, LG8	10–19	[135]
GWAS	132 advanced lines	SNP	AB4.1 QTL, 12 candidate genes	LG4	NR	[136]
Bi-parental	JG 62 × ICCV 05530 (188 RIL)	SSR, SNP	Two minor QTLs for seedling resistance, a minor QTL for adult plant resistance (*AB-Q-SR-4-1*, *AB-Q-APR-4-1*)	LG4	6.44–6.98	[137]
WGS	FLIP84-92C (2) × PI359075 (250 RILs), FLIP84-92C (3) × PI599072 (217 RILs)	SNP	*qABR4.1*, *qABR4.2*, *qABR4.3*; *CaAHL18* candidate gene	LG4	42	[138]
NGS-based BSA	ICCV 96029 × CDC Frontier (92 RILs), ICCV 96029 × Amit (139 RILs)	SNP	CPR01-qAB1.1, CPR01-qAB1.2, CPR01-qAB1.3, CPR01-qAB1.4, CPR01-qAB4.1, CPR01-qAB4.2, CPR01-qAB4.3, CPR01-qAB4.4, CPR01-qAB4.5, CPR01-qAB6.1, CPR01-qAB6.2, CPR01-qAB7.1	Ca1, Ca2, Ca4, Ca6, Ca7	NR	[139]
GBS	Amit × ICCV 96029 (133 RIL)	SNP	qAB2.1, qAB2.2, qAB2.3, qAB3.1, qAB4.1, qAB4.2, qAB5.1, qAB6.1 (8 QTLs); 5 candidate genes; *Ca2-ABAR*, *Ca2-PEI*, *Ca2-GDSL2*, *Ca4-ER2*, *Ca5-BTB*	Ca2, Ca3, Ca4, Ca5, Ca6	7–40	[140]
GBS	*C. arietinum* × *C. echinospermum* (134 RILs)	SNP	AB_echino_2014, AB_echino_2015	LG4	34–41	[141]
GBS	*C. arietinum* (GPF2) × *C. reticulatum* (ILWC 292) (187 RILs)	SNP	qab-4.1, qab-4.2, qab-7.1	LG4, LG7	7–11	[142]
GWAS	146 *C. reticulatum*, 44 *C. echinospermum*	SNP	WRKY TF (*Cr_02657.1*), (*Cr_09847.1*) encodes a TF or ARF family	LG3, LG4, LG6	6.7–15.2	[66]
GWAS	165 chickpea genotypes	SNP	11 R-QTL associated with resistance to specific pathotype, 6 R-QTL associated with resistance to two or more pathotypes	Ca1, Ca2, Ca6, Ca7	NR	[143]
GBS	AB_3279_ [ILC 3279 × ILC 1929], AB_482_ [ILC 482 × ILC 1929]	SNP	21 genomic regions, 9 newly identified genomic regions associated with AB resistance, 319 genes	CaLG02, CaLG04	11.2–39.3	[144]
GWAS	251 advanced breeding germplasm	SNP	26 genomic regions, at least 70 candidate genes (89 SNPs)	Ca1, Ca4, Ca6	NR	[145]
Bi-parental	*C. arietinum* × *C. reticulatum* [Gokce × Oyali-084 (160 F2:5)], *C. arietinum* × *C. echinospermum* [Gokce × Karab-092 (145 F2:5)]	SNP	4 QTL, 9 candidate genes (*Ca_10189*, *Ca_10186*, *Ca_05900*, *Ca_05898*, *Ca_05885*, *Ca_05884*, *Ca_03156*, *Ca_03143*, *Ca_03139*)	Ca2, Ca3, Ca6, Ca7	9.04–9.49	[121]
	Intraspecific (FLIP84–92C × PI359075), interspecific (FLIP84–92C × PI599072)	SNP	qABR4.1, qABR4.2, and qABR4.3; candidate genes *CaAP2* and *CaCNGCPD1*	Ca4	NR	[146]
GWAS	189 *C. arietinum*	SNP	19 SNPs	Ca1, Ca2, Ca3, Ca4, Ca7, Ca8		[147]
GBS	ILC 3279 × WR 315	SNP	4 Genomic regions, 30 genes from the identified regions were selected as robust candidates	Ca2, Ca4	NR	[148]
GWAS	219 chickpea lines	SNP	8 QTNs, 153 candidate genes	Ca1, Ca3, Ca4, Ca6, Ca7	NR	[58]
GWAS	mini-core germplasm of *C. reticulatum*	SNP	Two candidate genes (*Cr_14190.1_v2*, *Cr_14189.1_v2*)	Ca5	58	[149]
GBS	2790 chickpea lines	SNP	6 major QTLs	Ca1, Ca2, Ca3, Ca5, Ca7	33	[107]

NR = Not reported.

## Data Availability

No new data were created or analyzed in this study. Data sharing is not applicable to this article.
